# What Is the Hypoplastic Left Heart Syndrome?

**DOI:** 10.3390/jcdd10040133

**Published:** 2023-03-23

**Authors:** Robert H. Anderson, Adrian Crucean, Diane E. Spicer

**Affiliations:** 1 Biosciences Division, Newcastle University, Newcastle upon Tyne NE1 7RU, UK; 2 Department of Paediatric Cardiac Surgery, Birmingham Women’s and Children’s Hospital, Birmingham B4 6NH, UK; 3 Heart Institute, Johns Hopkins All Children’s Hospital, St. Petersberg, FL 33701, USA

**Keywords:** mitral atresia, mitral stenosis, aortic atresia, aortic stenosis, left ventricular hypoplasia

## Abstract

As yet, there is no agreed definition for the so-called “hypoplastic left heart syndrome”. Even its origin remains contentious. Noonan and Nadas, who as far as we can establish first grouped together patients as belonging to a “syndrome” in 1958, suggested that Lev had named the entity. Lev, however, when writing in 1952, had described “hypoplasia of the aortic outflow tract complex”. In his initial description, as with Noonan and Nadas, he included cases with ventricular septal defects. In a subsequent account, he suggested that only those with an intact ventricular septum be included within the syndrome. There is much to commend this later approach. When assessed on the basis of the integrity of the ventricular septum, the hearts to be included can be interpreted as showing an acquired disease of fetal life. Recognition of this fact is important to those seeking to establish the genetic background of left ventricular hypoplasia. Flow is also of importance, with septal integrity then influencing its effect on the structure of the hypoplastic ventricle. In our review, we summarise the evidence supporting the notion that an intact ventricular septum should now be part of the definition of the hypoplastic left heart syndrome.

## 1. Introduction

As yet, there is no agreement regarding the lesions that should be included within the so-called “hypoplastic left heart syndrome”. The definition by the International Nomenclature Society [1] specified that hearts having hypoplasia of the left ventricle in the setting of either transposition or double outlet right ventricle, or with a common atrioventricular junction, should be excluded. This criterion is important, since investigators seeking to establish the genetic background of the condition [2] have used mouse models with all these features, which should have resulted in their exclusion from consideration. The definition offered by the Nomenclature Society, however, makes no mention of the integrity of the ventricular septum. When the morphological features of potential candidates for inclusion are assessed, the findings point to the syndrome reflecting a spectrum of abnormality best interpreted as an acquired disease of fetal life, with an intact ventricular septum being an essential part of the anatomical make-up [3,4]. The left ventricle, of course, can also be hypoplastic when there is deficient ventricular septation. The hearts with ventricular septal deficiency, however, have markedly different phenotypic features from those found when the septum is intact. We suggest, therefore, that the lesions to be included within the syndrome should not only have essentially concordant atrioventricular and ventriculo-arterial connections, but also an intact ventricular septum.

## 2. The Historical Background to the Syndrome

According to Noonan and Nadas, it was Lev who introduced the term “hypoplastic left heart syndrome” [5]. This is not true. Lev had grouped together a series of hearts with “hypoplasia of the aortic tract complex” [6]. As far as we are able to establish, it was Noonan and Nadas themselves who first made reference to the potential grouping of the lesions with hypoplasia of the left ventricle as a “syndrome”. Their patients, all coming to autopsy, were grouped together on the basis of exhibiting the combination of “an obstructive lesion on the left side of the heart associated with hypoplasia of the left ventricle and right ventricular hypertrophy”. They excluded from consideration patients with “transposition”, which at the time would also have excluded those with double outlet right ventricle. They did include patients with both ventricular and atrioventricular septal defects. Of the 101 specimens making up their cohort, 70 exhibited only “hypoplasia of the aortic arch” in addition to the left heart hypoplasia. Of these, 37 had either ventricular or atrioventricular septal defects. Very few of the cases included by Noonan and Nadas [5], therefore, would now be included within the “syndrome” as defined using morphological criterions. Lev, in his own initial examination of hearts coming to autopsy because of “hypoplasia of the aortic outflow tract complex” [6], had also included examples with deficient ventricular septation. When he revisited the topic subsequent to the account provided by Noonan and Nadas, he restricted the cases to be included to those with an intact ventricular septum [7].

In their “guidelines” for treatment of infants born with the syndrome, the group convened by the European Associations for Paediatric Cardiology and Cardiothoracic Surgery had emphasised the phenotypic variability. They identified as subsets those with combined mitral and aortic atresia, those with mitral stenosis with aortic atresia, those with combined mitral and aortic stenosis, and those with the so-called hypoplastic left heart complex [8]. They also included an additional subset, namely those patients with mitral atresia and aortic stenosis in the setting of deficient ventricular septation. This is surprising, since like the Nomenclature Society [1], they had excluded those with double outlet right ventricle. It is individuals with the latter ventriculo-arterial connection, however, that make up a significant proportion of the subgroup better described as representing mitral atresia with a patent aortic root [9].

All of these discussions point to the need for a uniform definition for those individuals properly to be considered as belonging within the “syndrome”. Only when comparisons are made between comparable lesions will we finally unravel the genetic cues underscoring hypoplasia of the left ventricle, be it in the setting of an intact or deficient ventricular septum. We revisit here the developmental and morphological evidence supporting the notion that the lesions properly making up the syndrome, as opposed to the mere presence of left ventricular hypoplasia, are the consequence of an acquired disease of fetal life [3,4]. We do not deny that genetic cues are very likely to set the scene for such abnormal development. Whatever is responsible for the abnormality, the evidence to be presented shows that its effects do not become manifest until after closure of the embryonic interventricular communication.

## 3. Morphological Considerations

### 3.1. Evidence from Development

**Figure 1:**
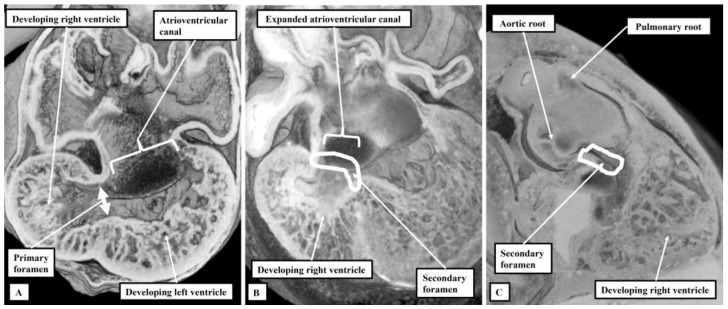
The images show the initial stages of formation of the ventricles in the human heart. Panel (**A**) shows a “four chamber” section at Carnegie stage 14, during the fifth week of development. The atrioventricular canal is supported exclusively by the developing left ventricle. Later in the fifth week, at Carnegie stage 15, as shown in panel (**B**), another four chamber section, the atrioventricular canal expanded to bring the cavity of the right atrium into communication with the developing right ventricle. This resulted in remodeling of the primary interventricular communication. As is shown in panel (**C**), also at Carnegie stage 15, but now showing the septal surface of the right ventricle, the secondary interventricular communication now provides the outlet for the developing left ventricle.

After formation and looping of the ventricular component of the developing heart, which occurred around 4 weeks after fertilisation in the human, and around the eleventh day of development in the mouse, the apical components of the developing right and left ventricles begun to balloon from the outer curvature of the ventricular loop (Figure 1A).

**Figure 2:**
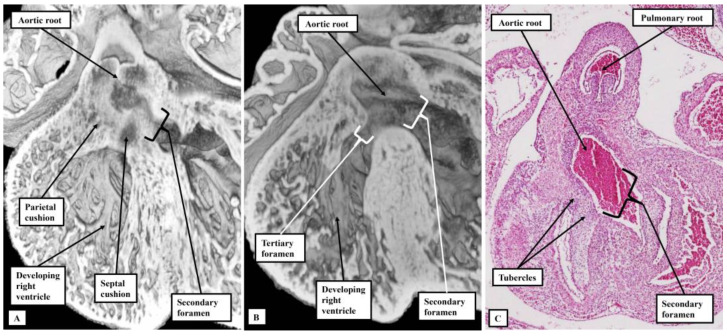
The images show the changes involved during transfer of the aortic root from the developing right to the developing left ventricle. Panels (**A**,**B**), in four chamber projection, are from the same mouse embryo sacrificed at embryonic day 13.5. Panel (**A**) shows how the proximal outflow cushions have fused to produce a partition at the base of the ventricle beneath the aortic root. Panel (**B**), a dorsal section from the same heart, shows how a tertiary foramen remains between the root and the cavity of the right ventricle. Panel (**C**), from a human embryo at Carnegie stage 21, shows how tubercles derived from the atrioventricular cushions close this tertiary foramen, becoming the membranous part of the ventricular septum.

At this initial stage, all the blood entering the developing left ventricle must pass through the interventricular communication to reach the developing right ventricle, which supports the entirety of the outflow tract. The first stage of ventricular remodeling is the expansion of the atrioventricular canal to provide the right ventricle with its own inlet (Figure 1B). The remodeling then leaves the interventricular communication providing the outlet for the left ventricle, since both arterial roots, by now separated one from the other, continue to be supported by the developing right ventricle (Figure 1C). The foramen is now a secondary entity. Ventricular remodeling is then completed by transfer of the aortic root from the right to the left ventricle. The embryo achieves this transfer by building a partition with the basal part of the right ventricle, fusing together the proximal cushions that have divided the outflow tract itself into the aortic and pulmonary channels (Figure 2).

When the partition is built at the ventricular base, sequestrating the cavity of the aortic root from the body of the right ventricle (Figure 2A), a communication remains between the root and the ventricle (Figure 2B). This is the tertiary interventricular foramen, although perhaps better described as an aorto-right ventricular communication. It is closed by formation and fusion of tubercles derived from the atrioventricular endocardial cushions (Figure 2C). This takes place during the seventh and eighth weeks of development in the human heart, and is complete by embryonic day 14.5 in the murine heart. The inference can be made that, if hearts are subsequently to develop left ventricular hypoplasia in the setting of an intact ventricular septum, then development must have proceeded in sufficiently normal fashion over the initial seven or eight weeks to permit formation of the membranous part of the septum. It is the stage of closure of the embryonic interventricular communication which marks the change from the embryonic to the fetal period of development. In those hearts with left ventricular hypoplasia along with an atrioventricular or ventricular septal defect, in contrast, the inference can be made that the progress of development was perturbed in the embryonic rather than the fetal period.

### 3.2. Morphological Findings in Postnatal Life

**Figure 3:**
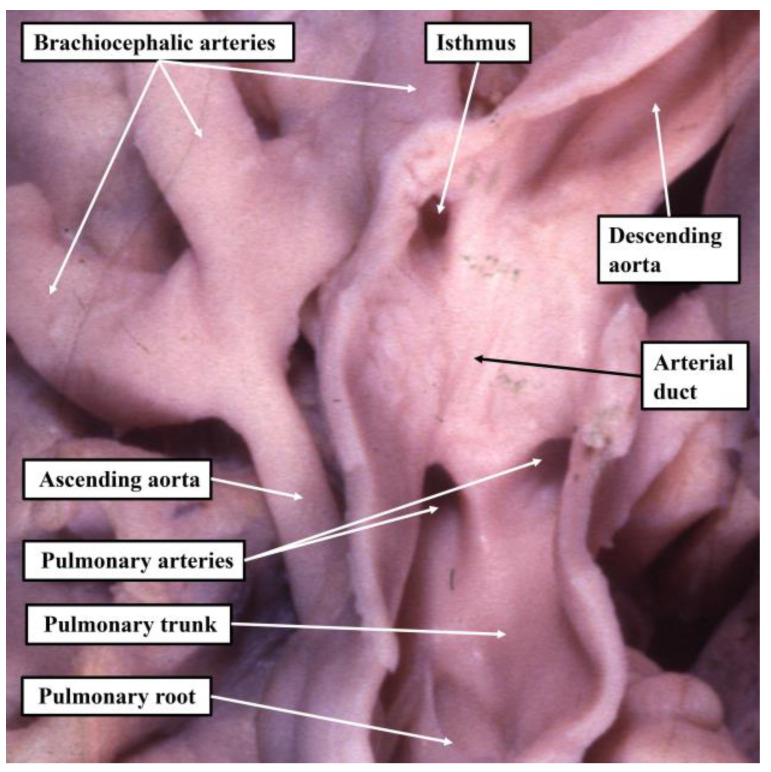
The image shows the characteristic feature of the intrapericardial arterial trunks and the extrapericardial pathways in the hearts grouped together by Lev as having hypoplasia of the aortic outflow tract complex. The major pathway from the heart is through the pulmonary trunk and the arterial duct. The aortic pathway is fed in retrograde fashion through the mouth of the aortic isthmus, which itself is encircled by ductal tissue.

The key feature of the circulation in the patients with left ventricular hypoplasia included in the original cohorts assembled by both Lev [6] and Noonan and Nadas [5] is the abnormal arrangement of the extrapericardial aortic pathway. The major pathway from the ventricles is from the pulmonary trunk, which continues as the arterial duct, with the transverse component of the aortic arch fed in retrograde fashion through the orifice of the aortic isthmus (Figure 3).

In the era prior to development of palliative surgical intervention [10], closure of the arterial duct would have cut off flow into the brachiocephalic and coronary arteries, producing the characteristic clinical features of the hypoplastic left heart syndrome as identified by Noonan and Nadas [5].

**Figure 4:**
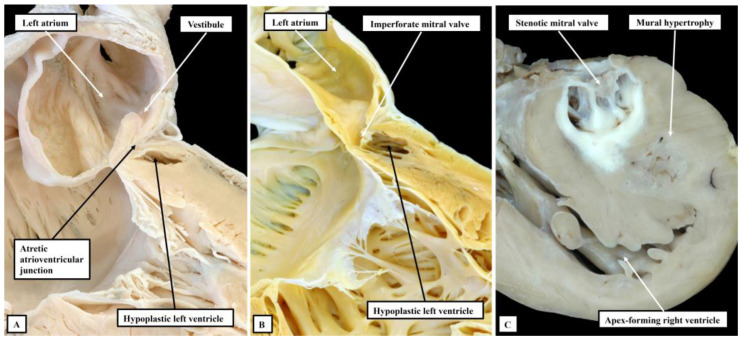
The images show the sequence of development of the mitral valve in the setting of hypoplastic left heart when the syndrome is defined on the basis of an intact ventricular septum. In panel (**A**), the atrioventricular connection had been formed, as evidenced by the presence of a blind-ending vestibule in the floor of the left atrium, but has become atretic. Panel (**B**) shows an imperforate mitral valve closing off a perforate left atrioventricular junction, while panel (**C**) shows a stenotic valve. Note that, in the setting of the stenotic valve, the cavity of the hypoplastic left ventricle is lined by a dense layer of fibroelastosis. Such elastosis is lacking in the hypoplastic left ventricles seen in panels (**A**,**B**).

Analysis of specimens collected together in the periods prior to development and widespread adoption of the Norwood sequence for surgical palliation [10] shows a spectrum of malformation of change in the setting of an intact ventricular septum [3,4]. These sequential changes are to be seen with regard to all the components of the left side of the heart when the left ventricle is hypoplastic in the setting of an intact ventricular septum. They are not seen in the same sequence when hypoplasia of the ventricle is found with either atrioventricular or ventricular septal defects. Thus, when the ventricular septum is intact, the mitral valve can be atretic, imperforate, or stenotic (Figure 4).

The presence of a perforate but stenotic mitral valve, of course, permits flow to take place into the left ventricle. It is almost certainly this flow into a restricted left ventricle that induces the formation of the dense layer of fibroelastosis that is characteristic for the ventricle when the mitral valve is stenotic and the ventricular septum is intact. Such an elastotic lining is not seen when the mitral valve is atretic or imperforate. It is similarly not usually found when the left ventricle is hypoplastic with either an atrioventricular or a ventricular septal defect. The features of the atretic, as opposed to imperforate, valve are also of interest. As already discussed, when first formed, the atrioventricular canal is supported exclusively by the developing left ventricle (Figure 1A). Presence of a connection between the left atrium and the left ventricle, therefore, is part and parcel of the normal make-up of the heart. At first sight, it seems that the arrangement with an atretic mitral valve represents absence of the left atrioventricular connection. The presence of a blind-ending vestibule within the floor of the left atrium, however, is suggestive of the fact that the connection had been formed during normal development, but had closed during the fetal period. The histological studies reported by Gittenberger-de-Groot and Wenink strongly support this progression of events [11].

**Figure 5:**
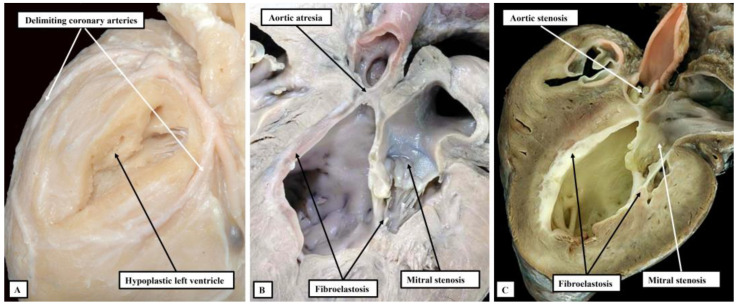
The images show the phenotypic features of the three subgroups of lesions that can be identified in the setting of hypoplasia of the left heart when the ventricular septum is intact. Panel (**A**) shows the grossly hypoplastic ventricle found when there is combined aortic and mitral atresia. The ventricle is no more than a slit on the diaphragmatic surface of the ventricular mass. It is found by dissecting between the delimiting coronary arteries. Panel (**B**) shows the features of aortic atresia with mitral stenosis, while panel (**C**) shows the findings with combined aortic and mitral stenosis. The left ventricle is larger in the setting of mitral stenosis, but is characterised by its dense fibroelastotic lining.

The overall findings from the hearts collected together in archives created prior to the advent of surgical correction support the existence of the phenotypical subsets highlighted in the European guidelines [8]. Thus, the hearts can be grouped together into subsets of combined aortic and mitral atresia, aortic atresia with mitral stenosis, and combined aortic and mitral stenosis (Figure 5).

**Figure 6:**
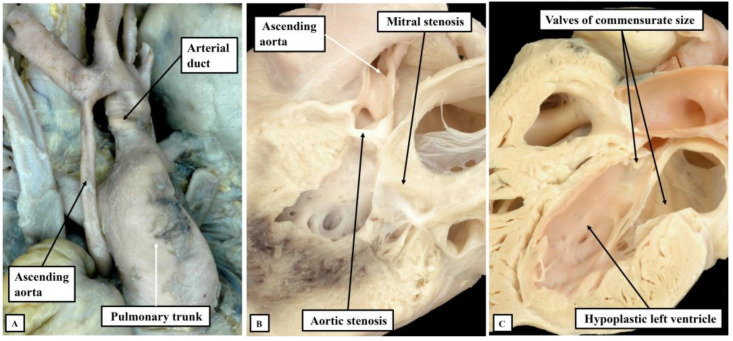
The images show the morphological features underscoring the notion that the hypoplastic left heart syndrome represents an acquired disease of fetal life, reflecting the time after the completion of ventricular septation that the left ventricle stopped its normal growth. Panel (**A**) shows the thread-like ascending aorta found when the aortic root is atretic. Panels (**B**) shows the larger aortic root found when the mitral valve is stenotic and the aortic valve is an imperforate shelf, with panel (**C**) showing the particularly well-formed root seen in the setting of the so-called hypoplastic left heart complex.

There is then an additional subset of lesions within the group of hearts having combined aortic and mitral stenosis. These hearts represent the best end of the spectrum of left ventricular hypoplasia found when the ventricular septum is intact. They have been nominated to represent a so-called “hypoplastic left heart complex” [12]. The hearts are important, since it is argued that, with this arrangement, biventricular surgical correction can be achieved simply by correcting the associated hypoplasia to be found in the aortic arch [12]. The hearts grouped in this fashion are characterised by the aortic and mitral valves being potentially stenotic, but commensurate in size with the proportions of the hypoplastic left ventricle. This can be interpreted as representing the best end of the spectrum of acquired disease of fetal life found when the left ventricle is hypoplastic in the presence of an intact ventricular septum (Figure 6).

## 4. Discussion

We have shown the morphological findings that underscore the inclusion of an intact ventricular septum as part of the definition of the hypoplastic left heart syndrome. Such a definition does not deny the fact that the left ventricle can be grossly hypoplastic when there is hypoplasia of the aortic arch in the settings of either an atrioventricular or ventricular septal defect. In the latter arrangements, however, in which the left atrioventricular valve is itself patent but stenotic, it is rare to find the hypoplastic left ventricle lined by fibroelastosis unless the defect itself is grossly restrictive. This fact then points to the influence of abnormal flow as instituting the fibroelastotic changes [13,14]. The morphological findings also point to the hearts included within the syndrome, as now suggested, as representing a spectrum of malformation. The severity of abnormality reflects the time during fetal life at which normal growth of the ventricle was perturbed. At the worst end of the spectrum, the inference can be made that the left ventricle ceased its normal growth early during the fetal period. In consequence, the mitral valve became atretic, as did the aortic root. These are the hearts with the smallest left ventricles, and with thread-like ascending aortas [3,15]. At the best end of the spectrum, reflecting cessation of growth much later during gestation, are the hearts that make up the so-called “hypoplastic left heart complex”. The aortic and mitral valves in these hearts are commensurate in size with that of the hypoplastic left ventricle. Patients with such hearts are potential candidates for biventricular surgical correction [12]. Recognition of the significance of the integrity of the ventricular septum is a vital feature for those hoping to identify the genetic background to hypoplasia of the left ventricle. Only when distinction is made between those with and without intact ventricular septums will it be feasible to understand the full significance of the genetic basis of the disease. As yet, the majority of mouse models alleged to represent the syndrome have failed to show the features of acquired fetal disease [2]. The production of the model implicating the importance of flow is now a step in the appropriate direction [16].

## 5. Conclusions

The morphological features of hearts found with hypoplasia of the left ventricle support dividing them into groups with and without an intact ventricular septum. The findings show that, when the ventricular septum is intact, the hearts can be interpreted as showing the features of an acquired disease of fetal life. For this reason, we recommend that the integrity of the ventricular septum should now be included as one of the defining features of the hypoplastic left heart syndrome.

## Data Availability

The data reviewed has previously been published in the works cited in our references.
